# Metastatic spinal tumor frailty index and New England spinal metastasis score show the most consistent performance for short-term postoperative outcomes: Single-center validation in 114 patients

**DOI:** 10.1016/j.xnsj.2026.100894

**Published:** 2026-04-25

**Authors:** Joseph A. Oldam, Mazen Taman, Weston C. de Lomba, Christian Schroeder, Owen P. Leary, Alexander A. Chernysh, Jonathan Arditi, Adetokunbo A. Oyelese, Jared S. Fridley, Tianyi Niu, Joaquin Q. Camara, Albert E. Telfeian, Ziya L. Gokaslan, Patricia L. Zadnik Sullivan

**Affiliations:** a Department of Neurosurgery, The Warren Alpert Medical School of Brown University, Providence, RI, United States; b Department of Neurosurgery, Rhode Island Hospital, Providence, RI, United States; c Department of Neurosurgery, The University of Texas at Austin Dell Medical School, Austin, TX, United States; d Department of Neurosurgery, UT Health Austin Spine and Nerve Center, TX, United States; e The Endoscopic Spine Institute Of New York, New York, NY, United States

**Keywords:** Spine, Frailty, Metastatic spinal disease, Perioperative risk, MSTFI, NESMS, Outcomes, Length of stay, Discharge disposition

## Abstract

**Background:**

Frailty indices are established tools for estimating long-term survival in oncology, yet their utility for predicting short-term surgical outcomes remains less defined. This study evaluates several frailty, comorbidity, and risk indices for predicting postoperative care needs and other short-term outcomes in patients undergoing spinal metastasis resection.

**Methods:**

A retrospective cohort study was performed on patients undergoing surgery for spinal metastasis at a tertiary spine center. Preoperative risk was assessed using the modified 5-item frailty index (mFI-5), Metastatic Spinal Tumor Frailty Index (MSTFI), modified Charlson Comorbidity Index (CCI), New England Spinal Metastasis Score (NESMS), modified Bauer score (mBauer), and Spinal Instability Neoplastic Score (SINS). Multivariate logistic regression evaluated associations between indices and nonroutine discharge, prolonged length of stay (LOS), and reoperation, as well as other secondary outcomes.

**Results:**

Among 114 patients (mean age 65.6±10.7 years; 57.9% male), 57.0% were discharged to nonroutine settings, 16.7% underwent reoperation, and the mean length of stay was 11.1±10.8 days. MSTFI was independently associated with nonroutine discharge (OR=2.81, 95% CI [1.64–4.84], p<.001) but not prolonged LOS (OR=1.51 [0.99–2.30], p=.055). NESMS was also associated with nonroutine discharge (OR=0.49 [0.28–0.87], p=.015). Secondary analyses identified associations of NESMS spine-related complications (OR=0.46 [0.24–0.87], p=.017) and 90-day mortality (OR=0.48 [0.23–0.98], p=.045). No index significantly predicted reoperation. ROC analysis demonstrated that MSTFI and NESMS outperformed other indices for nonroutine discharge and prolonged LOS; DeLong’s were equivocal.

**Conclusions:**

In this single-center surgical cohort, MSTFI and NESMS were the most consistent risk-stratification tools for short-term outcomes, particularly discharge disposition and LOS. Secondary analyses suggested additional associations of NESMS with spine-related complications and 90-day mortality, whereas no index reliably predicted reoperation. Incorporating MSTFI and/or NESMS into preoperative assessment may improve risk stratification and perioperative planning.

## Background

Frailty, characterized by diminished physiological reserve and increased vulnerability to stress, has been consistently linked to poor postoperative outcomes, including complications, prolonged length of stay (LOS), higher costs, and mortality across surgical specialties [[1], [2], [3], [4], [5], [6], [7]]. It is common among older adults, affecting approximately 10% of individuals over 65 and up to half of those over 85, and is closely associated with multimorbidity, disability, and sarcopenia [8]. Despite its growing role in perioperative risk assessment, frailty remains underutilized in spine oncology, where patients with metastatic spinal disease (MSD) face especially high complication rates [9].

The spine is the most frequent site of skeletal metastasis, and spinal involvement occurs in a substantial proportion of patients with advanced cancer [10,11]. Surgical resection remains a primary treatment option for patients with mechanical and/or neurologic instability, aiming to relieve pain and preserve neurologic function and mobility [[12], [13], [14]]. Nevertheless, outcomes remain poor: reported complication rates range from 10% to 52%, and up to 13% of patients die within 30 days of surgery [12,13,15,16]. Selecting patients who are most likely to benefit from surgical therapy among patients with spinal metastases is challenging, especially for patients with intermediate spinal instability as evaluated by the Spinal Instability Neoplastic Score (SINS) [17,18]. More accurate tools to identify patients at highest risk for adverse outcomes are therefore essential to inform surgical decision-making and optimize perioperative care.

### Study rationale and objectives

Despite their promise, frailty assessments are not yet fully integrated into MSD management. Frailty can be measured by a series of previously validated indexes which each capture slightly different information, including two scores specifically designed for use in spinal metastasis patients. The metastatic spinal tumor frailty index (MSTFI) is one score that aims to predict perioperative risks in MSD surgery while the New England Spinal Metastasis Score (NESMS) is another score that aims to estimate survival prognosis in patients with MSD to guide clinical decision making [13,19,20]. Furthermore, there exists limited evidence linking specific indices to outcomes of particular interest to spine surgeons treating patients with MSD, such as complications, LOS, or nonroutine discharge [21]. This study addresses these gaps by evaluating the predictive performance of several commonly used frailty and comorbidity indices in patients undergoing surgical resection for MSD. Validating these tools in this population has the potential to enhance preoperative counseling, guide perioperative planning, and ultimately improve patient outcomes. As such, this study provides a head-to-head evaluation of commonly used frailty indices in patients undergoing MSD surgery, with a particular focus on length of stay and discharge disposition in an independent single-center cohort of surgically treated patients in which all scores were applied without modification or refitting.

## Methods

### Data collection

A retrospective review was conducted of patients who underwent surgical resection for spinal metastases at a comprehensive spine center between April 2015 and May 2023. The study was approved by the institutional review board with a waiver of informed consent, and all data were handled in accordance with ethical standards and confidentiality guidelines. Included patients were ≥18 years old. Data abstraction from electronic medical records included demographics, comorbidities, preoperative treatments, operative details, and postoperative outcomes. Frailty was assessed using the modified 5-item frailty index (mFI-5) [22], MSTFI [19], modified Charlson comorbidity index (CCI) [23], NESMS [13], SINS [17,18], and modified Bauer score (mBauer) [24]. For each, scoring followed the original publications and was calculated from preoperative data closest to the date of surgery.

### Outcomes

Primary outcomes included reoperation, defined as any unplanned return to the operating room for a complication at any time during follow-up; prolonged length of stay, defined as hospitalization exceeding the 75th percentile for the cohort (>14 days); and nonroutine discharge, defined as any discharge disposition other than home. Secondary outcomes included spine-related and medical complications; 30-day emergency department visits; readmission, defined as any hospital readmission within follow-up regardless of cause; and 90-day mortality. Spine-related complications encompassed seroma, dehiscence, surgical site infection, cerebrospinal fluid leak or pseudomeningocele, graft or cage subsidence or extrusion, construct or instrumentation instability with or without breakage, additional or progressing neoplastic disease at the same or a different spinal level, and new neurologic deficits unrelated to planned neurological sacrifice. Medical complications encompassed myocardial infarction, pulmonary embolism, deep vein thrombosis, cerebrovascular accident or stroke, new-onset neuropathic pain, delirium, dysphagia, anemia, pneumonia, urinary tract infection, and other unspecified medical complications. Although all outcomes were analyzed using a common statistical framework to facilitate comparison across indices, these endpoints differ in timing and underlying mechanisms, with frailty expected to exert a stronger influence on recovery-related outcomes (eg, discharge disposition and length of stay) than on outcomes driven predominantly by surgical or oncologic factors.

### Statistical analysis

Descriptive statistics were generated for all baseline covariates and outcomes. Multivariable logistic regression models were fitted to evaluate the predictive performance of each frailty or comorbidity index (mFI-5, CCI, mBauer score, SINS, MSTFI, NESMS) for each primary and secondary outcome, adjusting for age, sex, body mass index, hypertension, diabetes, tobacco use, prior radiation, procedure duration, and junctional spinal region. These factors were adjusted for because they are also hypothesized to influence the outcomes of interest. Events-per-variable (EPV) ratios were calculated for each model to assess model stability and risk of overfitting.

Given the low number of reoperation events, we used Firth-penalized logistic regression. For outcomes with fewer than 30 events (eg, prolonged length of stay, 90-day mortality), logistic regression with ridge penalization was applied, and internal validation was performed using 1,000 bootstrap resamples. Discrimination was quantified using receiver operating characteristic (ROC) curves and the area under the curve (AUC) with 95% confidence intervals. DeLong’s test was used to compare AUCs between MSTFI and NESMS for each outcome. Model calibration was assessed using bootstrap-corrected calibration plots, calibration slope, and the Brier score.

Decision curve analysis (DCA) was performed for each primary outcome (nonroutine discharge, prolonged LOS, reoperation) across prespecified threshold ranges set *a priori* from institutional experience. For nonroutine discharge, thresholds from 0.30 to 0.80 were selected, reflecting the clinical range where clinicians might intervene with early discharge planning. For prolonged LOS, thresholds of 0.20–0.60 were used, corresponding to probabilities at which additional perioperative resources (eg, rehabilitation, social work) might be mobilized. For reoperation, a narrower threshold range of 0.05–0.30 was applied, reflecting the lower expected prevalence of this outcome and the lower probability at which a surgeon might consider modifying the operative plan. Curves were compared against “treat all” and “treat none” strategies, with net benefit reported at each threshold. In sensitivity analysis, DCA was repeated across alternative threshold probability grids spanning lower and higher ranges around prespecified intervals (Supplementary Fig. S2; Supplementary Table S2).

All analyes were performed using R (version 4.4.1; R Foundationfor Statistical Computing, Vienna, Austria) and RStudio (version 2024.04.2+764; Posit Software, PBC, Boston, Massachusetts, USA. There were no missing data for variables used in multivariable models; preoperative ASIA grade was occasionally undocumented, was not retrospectively inferred, and was summarized using available-case denominators. Statistical significance was set at p<.05.

## Results

### Patient demographics and clinical characteristics

The study cohort consisted of 114 patients (mean age 65.6±10.7 years; 57.9% male). Mean BMI was 27.1±5.8. Hypertension was present in 55.3%, and 21.9% had diabetes (2.6% IDDM, 19.3% NIDDM). Smoking history included 33.3% never, 23.7% current, and 43.0% former smokers. Radiation exposure was reported in 34.2%. Operative course was heterogenous but predominantly involved posterior approach with extensive decompression and many instrumented levels. Mean procedure duration was 406.8±163.7 minutes, with semi-rigid, mobile, and junctional spinal locations accounting for 45.6%, 16.7%, and 37.7% of cases, respectively. Full baseline, operative, functional, and frailty characteristics are described in greater detail in Table 1, Table 2. Postoperative outcomes are given in Table 3.

**Table 1:** Patient demographics and clinical characteristics (N=114).

Characteristic	Value
**Age, years**	65.6±10.7
**Sex**	
Male	66 (57.9)
Female	48 (42.1)
**Body mass index, kg/m²**	27.1±5.8
**Comorbidities**	
Hypertension	63 (55.3)
Diabetes mellitus	25 (21.9)
NIDDM	22 (19.3)
IDDM	3 (2.6)
**Smoking history**	
Never	38 (33.3)
Current	27 (23.7)
Former	49 (43.0)
**Prior radiation**	39 (34.2)

Values are presented as counts (percentages) or mean ± standard deviation, as appropriate.

NIDDM, non–insulin-dependent diabetes mellitus; IDDM, insulin-dependent diabetes mellitus.

**Table 2:** Functional, frailty, and operative characteristics of the study cohort (N=114).

Characteristic	Value
**Preoperative ECOG**	
0	17 (14.9%)
1	43 (37.7%)
2	24 (21.1%)
3	22 (19.3%)
4	8 (7.0%)
**Preoperative ASIA**	
C	6 (5.3%)
D	4 (3.5%)
E	65 (57.0%)
**Frailty/Comorbidity Indices**	
mFI-5	1.1±0.9
MSTFI	1.8±1.2
Modified CCI	3.1±1.8
mBauer score	1.3±1.0
NESMS	0.9±0.9
SINS	9.6±3.0
**SINS location**	
Semi-rigid	52 (45.6%)
Mobile	19 (16.7%)
Junctional	43 (37.7%)
Not recorded	39 (34.2%)
**Approach**	
Posterior only	62 (54.4%)
Anterior only	3 (2.6%)
Combined/staged anterior-posterior	49 (43.0%)
**Levels of exposure, median [IQR]**	6 [3–8]
**Fusion/fixation performed**	83 (72.8%)
**Any decompression performed**	91 (79.8%)
**Levels decompressed, median [IQR]**	3 [1–3]
**Procedure duration, minutes**	406.8±163.7
**Estimated blood loss, mL, median [IQR]**	500 [250–950]

Spinal Instability Neoplastic Score (SINS) locations were most frequently semi-rigid (45.6%) or junctional (37.7%). Functional status was assessed using Eastern Cooperative Oncology Group (ECOG) performance status and the American Spinal Injury Association (ASIA) Impairment Scale. Frailty and comorbidity were summarized using multiple validated indices. Values are presented as counts (percentages) or mean ± standard deviations unless otherwise specified.

SINS, Spinal Instability Neoplastic Score; ECOG, Eastern Cooperative Oncology Group; ASIA, American Spinal Injury Association; mFI-5, modified 5-item frailty index; MSTFI, Metastatic Spinal Tumor Frailty Index; CCI, Charlson Comorbidity Index; mBauer, modified Bauer score; NESMS, New England Spinal Metastasis Score; IQR, interquartile range.

**Table 3:** Postoperative outcomes (N=114).

Primary outcomes	Value
Nonroutine discharge	65 (57.0%)
Prolonged LOS	25 (21.9%)
Reoperation for complication	19 (16.7%)
**Secondary Outcomes**	
Medical complication	33 (28.9%)
Spine-related complication	35 (30.7%)
30-day ED visit	39 (34.2%)
Readmission within follow-up	27 (23.7%)
90-day mortality	25 (21.9%)

Primary outcomes included nonroutine discharge (any discharge other than home), prolonged length of stay (LOS; >14 days, corresponding to the 75th percentile of the cohort), and reoperation for complication at any time during follow-up. Secondary outcomes included medical complications, spine-related complications, 30-day emergency department (ED) visits, readmission within follow-up, and 90-day mortality.

LOS, length of stay; ED, emergency department.

### Primary outcomes

Among recovery-oriented outcomes most plausibly influenced by physiologic reserve in this surgically selected cohort—particularly nonroutine discharge and prolonged length of stay—MSTFI and NESMS were the most consistent risk-stratification tools in this cohort, though these endpoints also overlap conceptually with index components (Table 4). For nonroutine discharge, higher MSTFI was strongly associated with increased risk (OR 2.81; 95% CI 1.64–4.84; p<.001), whereas higher NESMS was protective (OR 0.49; 95% CI 0.28–0.87; p=.015). Both indices demonstrated good discrimination (AUC 0.84 and 0.78, respectively); DeLong’s test was not signficant (ΔAUC=0.06, p=.095). Calibration supported these findings, with Brier scores of 0.161 (MSTFI) and 0.182 (NESMS). ROC curve analysis revealed that both indices yielded a greater net clinical benefit for discharge prediction across a broad range of threshold probabilities compared with treating all or no patients as high-risk. For prolonged LOS, MSTFI (OR 1.51; 95% CI 0.99–2.30; p=.055) and NESMS (OR 0.49; 95% CI 0.24–1.00; p=.051) had similar discriminative performance (AUCs: 0.78–0.79; DeLong’s: p=.60). Both indices showed favorable calibration (Brier ∼0.15) among evaluated indices, and DCA again suggested clinical utility. No frailty index significantly predicted reoperation, though MSTFI and NESMS both achieved moderate discrimination (AUCs ∼0.76–0.78). MSTFI and NESMS ROC plots for nonroutine discharge and prolonged LOS are shown in the Figure. Overall, MSTFI and NESMS generally showed stronger discrimination than other frailty measures for discharge disposition and LOS, whereas performance for reoperation was limited. DCA demonstrated net benefit for MSTFI and NESMS robust to multiple threshold ranges for nonroutine discharge and prolonged LOS, but not reoperation (Supplementary Fig. S2).

**Table 4:** Performance of frailty index by outcome.

Outcome	OR (95% CI)	p-value	AUC (95% CI)
**Nonroutine discharge**			
MSTFI	2.81 (1.64–4.84)	**<.001**	0.84 (0.77–0.84)
NESMS	0.49 (0.28–0.87)	.015	0.78 (0.69–0.78)
mFI-5	0.78 (0.32–1.92)	.595	0.74 (0.65–0.74)
CCI	0.76 (0.49–1.18)	.216	0.75 (0.66–0.75)
mBauer	1.24 (0.75–2.06)	.401	0.75 (0.66–0.75)
SINS	0.97 (0.83–1.14)	.743	0.74 (0.65–0.74)
**Prolonged LOS**			
MSTFI	1.51 (0.99–2.30)	.055	0.78 (0.69–0.78)
NESMS	0.49 (0.24–1.00)	.051	0.79 (0.71–0.79)
mFI-5	0.96 (0.34–2.73)	.933	0.76 (0.66–0.76)
CCI	1.12 (0.67–1.87)	.671	0.77 (0.67–0.77)
mBauer	1.12 (0.63–2.02)	.698	0.76 (0.65–0.76)
SINS	0.88 (0.73–1.06)	.174	0.78 (0.69–0.78)
**Reoperation**			
MSTFI	0.93 (0.62–1.39)	.715	0.76 (0.67–0.76)
NESMS	0.67 (0.35–1.29)	.231	0.78 (0.69–0.78)
mFI-5	0.91 (0.35–2.37)	.843	0.76 (0.67–0.76)
CCI	0.77 (0.46–1.30)	.332	0.77 (0.68–0.77)
mBauer	1.41 (0.79–2.50)	.244	0.78 (0.69–0.78)
SINS	0.90 (0.75–1.09)	.282	0.77 (0.68–0.77)

Odds ratios (OR) with 95% confidence intervals (CI) are adjusted for age, sex, body mass index, hypertension, diabetes, tobacco use, prior radiation, procedure duration, and junctional spinal region. Reoperation models used Firth-penalized logistic regression due to the limited number of events. Prolonged length of stay (LOS, >14 days) models used ridge-penalized logistic regression with bootstrap validation (1,000 resamples). Discrimination was assessed using area under the receiver operating characteristic curve (AUC). LOS, length of stay; MSTFI, Metastatic Spinal Tumor Frailty Index; NESMS, New England Spinal Metastasis Score; mFI-5, modified 5-item Frailty Index; CCI, Charlson Comorbidity Index; mBauer, modified Bauer score; SINS, Spinal Instability Neoplastic Score.

**Figure:**
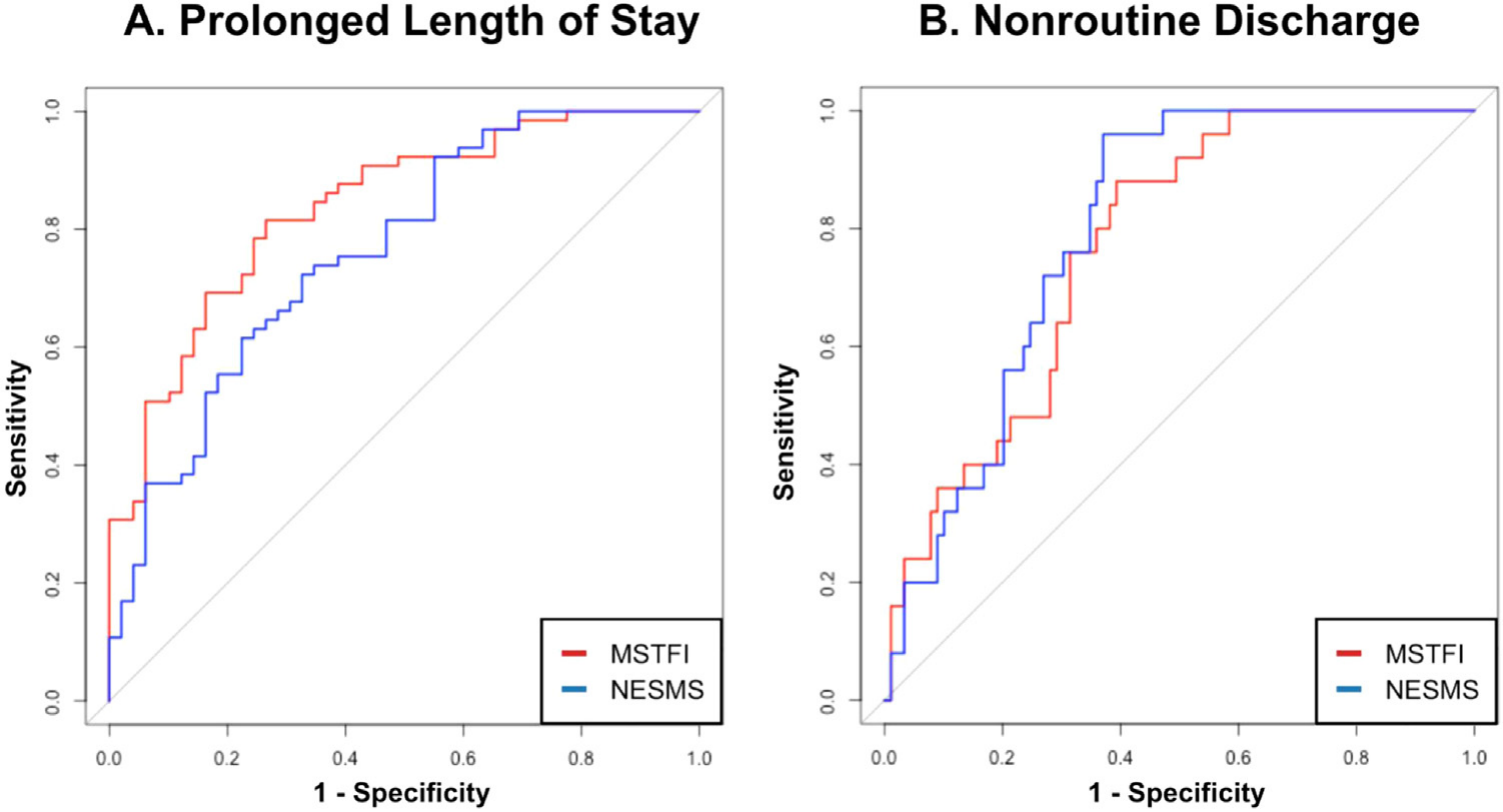
Receiver operating characteristic (ROC) curves of the multivariate logistic regression models used to predict the effect of various frailty assessments on prolonged length of stay (A) and nonroutine discharge (B). For nonroutine discharge, area under the curve (AUC) values were 0.84 and 0.78 for MSTFI and NESMS, respectively. For prolonged length of stay, AUC values were 0.78 and 0.79 for NESMS and MSTFI, respectively.

### Secondary outcomes

In secondary analyses, lower NESMS was significantly associated with spine-related complications (OR 0.46; 95% CI 0.24–0.87; p=.017) and 90-day mortality (OR 0.48; 95% CI 0.23–0.98; p=.045). MSTFI was in medical complications and ED visits but was not significant. Other indices, including mFI-5, CCI, mBauer, and SINS, were not consistently predictive across outcomes and generally showed lower discrimination and higher Brier scores. Detailed model performance characteristics are provided in Table 5.

**Table 5:** Performance of frailty index by secondary outcome.

Any medical complication	OR (95% CI)	p-value	AUC (95% CI)
MSTFI	1.33 (0.93–1.92)	.119	0.70 (0.60–0.70)
NESMS	0.67 (0.38–1.20)	.178	0.69 (0.58–0.69)
mFI-5	0.98 (0.40–2.36)	.956	0.67 (0.56–0.67)
CCI	0.59 (0.34–1.04)	.069	0.70 (0.60–0.70)
mBauer	0.82 (0.49–1.36)	.440	0.68 (0.56–0.68)
SINS	1.13 (0.96–1.34)	.148	0.71 (0.60–0.71)
**Any spine complication**			
MSTFI	1.19 (0.81–1.74)	.373	0.71 (0.60–0.71)
NESMS	0.46 (0.24–0.87)	**.017**	0.73 (0.63–0.73)
mFI-5	0.64 (0.24–1.70)	.367	0.70 (0.60–0.70)
CCI	1.07 (0.69–1.66)	.774	0.70 (0.59–0.70)
mBauer	0.56 (0.31–0.99)	**.046**	0.73 (0.63–0.73)
SINS	0.98 (0.83–1.15)	.802	0.69 (0.58–0.69)
**30-day ED visit**			
MSTFI	1.23 (0.86–1.76)	.259	0.72 (0.62–0.72)
NESMS	1.03 (0.60–1.77)	.925	0.73 (0.62–0.73)
mFI-5	0.41 (0.15–1.10)	.077	0.75 (0.65–0.75)
CCI	0.96 (0.63–1.46)	.855	0.73 (0.63–0.73)
mBauer	0.58 (0.34–1.00)	**.049**	0.75 (0.65–0.75)
SINS	1.07 (0.92–1.25)	.390	0.73 (0.63–0.73)
**Readmission**			
MSTFI	1.10 (0.73–1.67)	.645	0.76 (0.66–0.76)
NESMS	0.62 (0.32–1.21)	.160	0.77 (0.67–0.77)
mFI-5	0.71 (0.25–2.04)	.530	0.76 (0.66–0.76)
CCI	0.83 (0.49–1.38)	.465	0.76 (0.66–0.76)
mBauer	0.85 (0.46–1.56)	.594	0.76 (0.66–0.76)
SINS	1.00 (0.84–1.19)	.985	0.76 (0.65–0.76)
**90-day mortality**			
MSTFI	1.23 (0.80–1.88)	.346	0.75 (0.65–0.75)
NESMS	0.48 (0.23–0.98)	**.045**	0.79 (0.69–0.79)
mFI-5	1.21 (0.43–3.43)	.715	0.75 (0.65–0.75)
CCI	0.72 (0.39–1.35)	.312	0.76 (0.66–0.76)
mBauer	1.14 (0.65–1.98)	.646	0.75 (0.64–0.75)
SINS	1.02 (0.85–1.23)	.829	0.76 (0.65–0.76)

Odds ratios (OR) with 95% confidence intervals (CI) are adjusted for age, sex, body mass index, hypertension, diabetes, tobacco use, prior radiation, procedure duration, and junctional spinal region. Standard logistic regression was applied unless <30 outcome events, in which case ridge-penalized regression with bootstrap validation (1,000 resamples) was used. Discrimination was assessed using area under the receiver operating characteristic curve (AUC).

MSTFI, Metastatic Spinal Tumor Frailty Index; NESMS, New England Spinal Metastasis Score; mFI-5, modified 5-item Frailty Index; CCI, Charlson Comorbidity Index; mBauer, modified Bauer score; SINS, Spinal Instability Neoplastic Score.

## Discussion

### Frailty indices of choice for metastatic spine tumors: MSTFI and NESMS

Frailty assessments are increasingly examined for their ability to predict recovery and care needs following surgical resection of MSD, rather than for outcomes driven primarily by surgical complexity or oncologic progression. Among the indices analyzed, MSTFI and NESMS (two scores explicitly designed for use in this patient population) demonstrated superior predictive performance for prolonged LOS and nonroutine discharge in this single-center operative cohort. The MSTFI incorporates multidimensional parameters such as anemia, chronic lung disease, coagulopathy, malnutrition, and surgical approach [21,25]. Similarly, NESMS considers multiple factors such as ambulatory status, serum albumin levels, and the primary tumor type and metastatic burden through the Modified Bauer Score [[24], [25], [26]]. Though in no way causal, and with some conceptual overlap between index components and recovery-oriented endpoints, the ability of these frailty assessments to integrate diverse frailty factors likely accounts for their strong predictive performance. Because this surgically treated cohort is both selected and operatively heterogeneous, and these factors are not fully modeled, these findings are best interpreted as comparative risk stratification within an operative context rather than evidence that any frailty index can independently outperform surgical complexity or oncologic burden.

Overall, our findings are directionally consistent with prior work examining the utility of MSTFI and NESMS for recovery-oriented endpoints such as nonroutine discharge and prolonged LOS [11,13,15]. Associations with complications or mortality remain limited and exploratory [25]. While MSTFI achieved the highest AUC for nonroutine discharge in our cohort, the difference from NESMS was modest and DeLong testing did not support a clear discriminatory advantage. Accordingly, these findings are best interpreted as showing broadly similar performance of the two indices for recovery-oriented endpoints, rather than a clinically decisive advantage of one over the other. Prognostic utility with endpoints driven by surgical complexity remains limited, highlighting the need for larger datasets that can better distinguish frailty from tumor-specific biology and treatment context across metastatic spine subgroups [27].

Importantly, calibration metrics (Brier scores ∼0.16–0.18) and DCA suggest that both MSTFI and NESMS may offer net clinical benefit for anticipatory planning within the operative decision context, such as discharge arrangements and perioperative counseling. Similar utility was not observed for outcomes more directly influenced by surgical or oncologic factors. Across clinically relevant threshold ranges (10%–50%), both indices achieved positive net benefit compared to “treat all” or “treat none” approaches. While strict threshold-based decision making is not supported by the evidence, results do suggest that these scores offer utility in identifying higher-risk patients for additional perioperative support as necessary and for anticipating the discharge needs of these patients. Especially when weighing the potential risks of surgery for intermediate SINS patients, these indices may help surgeons make more informed decisions.

### Charlson comorbidity index (CCI)

While MSTFI and NESMS led other indices with regard to predicting LOS and nonroutine discharge, the CCI did not significantly predict any outcomes, such as complications, reoperation, or general disposition. Nonetheless, the CCI captures age and a wide range of comorbidities (cardiac, pulmonary, neurological, oncological, hematological, etc.) comprising 17 weighted variables, with higher scores generally corresponding with worse morbidity and mortality risk [28]. Although the CCI is not disease-specific, its weighting structure captures overall physiological burden, which may partly overlap with the frailty constructs represented by MSTFI and NESMS [28,29]. While prior studies have similarly linked CCI to reoperation risk in spine and cranial surgery [30,31], consistent with other reports, its predictive capacity appears limited compared to indices tailored for oncologic spinal populations [32].

### mFI-5 and mBauer

With similar comorbidities to CCI, neither mFI-5 nor mBauer demonstrated consistent predictive value in this cohort. The mFI-5, although widely used as a general frailty index, captures a narrower set of comorbidities and may underestimate frailty in cancer patients, especially when nutritional or oncologic variables drive risk [20,21]. Similarly, the mBauer score provided limited prognostic information outside of its original oncologic context. Among our MSD cohort, these findings underscore the importance of cancer-specific indices such as NESMS and MSTFI, which better capture tumor biology in frailty estimates.

### Clinical implications

Incorporating frailty indices such as MSTFI and NESMS into preoperative evaluations can help identify patients at risk for prolonged hospitalization and nonroutine discharge. Early recognition of frailty creates an opportunity to implement prehabilitation programs that address physical fitness, nutrition, and psychological readiness for patients who require surgical stabilization for MSD [4]. Just as importantly, these tools can guide conversations with patients and families by setting realistic expectations about LOS and the likelihood of rehabilitation or extended care after surgery [33]. This type of counseling ensures that surgical decisions are aligned with patient goals and may, in some cases, support consideration of nonsurgical or palliative options such as radiotherapy or systemic therapy when the anticipated recovery does not match those goals.

From a systems standpoint, better predicting discharge needs allows earlier involvement of multidisciplinary teams (including physical and occupational therapy, case management, and social work) to build tailored discharge plans. By conferring the capacity to clarify expectations and improve care coordination, frailty assessments have the potential to reduce complications related to poor transitions of care. This hypothesized benefit may indirectly translate to lower readmission risk, though this requires further study [6,7,34,35]. Overall, MSTFI and NESMS appear to offer similar practical utility for surgical planning and communication in this operative MST population.

Finally, it is worth noting that these metrics could be calculated automatically from data routinely included in the electronic medical record. Future documentation augmentation within the EMR or “frailty alert” functions that flag at-risk patients to raise provider awareness could make the practical implementation of these tools even easier.

### Limitations

Limitations of prognostic indices in metastatic spine disease—particularly frailty-based tools (eg, MSTFI and NESMS)—show heterogeneous performance across cohorts and endpoints and omit potentially important domains such as body composition/sarcopenia [25]. Because these indices incorporate variables proximal to recovery (eg, anemia or nutrition proxies, functional status, tumor burden), associations with LOS and discharge may partly reflect conceptual overlap and are therefore best interpreted as pragmatic risk stratification rather than causal effects. Several granular drivers of outcomes (eg, extent of decompression, number of instrumented levels, surgical approach, systemic oncologic status) were not modeled and may confound observed relationships; indices may partially proxy operative aggressiveness or disease burden. Despite the likely importance of histology and malignancy potential in shaping postoperative risk, the present dataset does not permit more granular evaluation across primary tumor subtypes. Finally, this retrospective single-center cohort was limited to surgically treated patients, excluding patients deemed too frail for surgery and truncating the frailty spectrum. Accordingly, discrimination, calibration, and decision-curve findings should be interpreted within an operative decision context, as institutional case-mix, surgical selection, perioperative management, and discharge practices may limit generalizability.

### Strengths of the present study

This study offers several strengths that extend prior work on frailty assessment in MSD. First, we provide a single-center validation in an independent cohort using granular, chart-level data among a limited set of indications to address limitations noted in early MSTFI and NESMS derivation studies that relied on national databases [13,19]. Second, unlike prior analyses that typically compared only one or two indices at a time [26,32], we directly evaluate six commonly used frailty, comorbidity and clinical risk measures (MSTFI, NESMS, mFI-5, CCI, mBauer, SINS) within the same contemporary surgical cohort to establish the most cohesive head-to-head comparison of short-term outcomes in the literature. Third, we focus on short-term, clinically actionable outcomes (particularly nonroutine discharge and prolonged LOS) that have been underrepresented in earlier MSTFI and NESMS works, which emphasize mortality or broad complication categories [12,13]. Finally, we apply more rigorous performance assessment than most prior validations by incorporating penalized regression, bootstrap-corrected calibration, Brier scores, and decision-curve analysis as methodological enhancements, as recommended in recent critical appraisals of frailty indices [20,25].

## Conclusion

This single-center surgical cohort study highlights the utility of frailty assessments in predicting near-term postoperative outcomes in patients undergoing surgical resection for spinal metastases. Among the indices evaluated, MSTFI and NESMS were the most consistent risk-stratification tools for recovery-oriented short-term postoperative outcomes, particularly for nonroutine discharge and prolonged LOS. Our findings underscore the importance of incorporating frailty assessments into preoperative evaluations to inform risk-benefit discussions and optimize patient management. Larger prospective metastatic spine registries with greater histologic granularity may clarify how frailty—and tumor biolog—jointly influence postoperative outcomes and inform future integration of frailty indices into spinal surgical oncology guidelines.

## Author contributions

**Joseph Oldam:** Conceptualization; Data Curation, Investigation, Validation, Writing—Original Draft, Writing—Review and Editing. **Mazen Taman:** Conceptualization; Data Curation, Investigation, Validation, Writing—Original Draft. **Weston de Lomba:** Conceptualization; Data Curation; Formal Analysis, Methodology, Software, Visualization, Writing—Original Draft, Writing—Review and Editing. **Christian Schroeder:** Conceptualization, Data Curation, Investigation, Project Administration. **Owen Leary:** Data Curation, Project Administration, Writing—Original Draft, Writing—Review and Editing. **Alexander Chernysh:** Data Curation, Investigation. **Jonathan Arditi:** Data Curation, Investigation. **Adetokunbo Oyelese:** Supervision, Resources, Writing—Review and Editing. **Jared Fridley:** Supervision, Resources, Writing—Review and Editing. **Tianyi Niu:** Supervision, Resources, Writing—Review and Editing. **Joaquin Camara:** Supervision, Resources, Writing—Review and Editing. **Albert Telfeian:** Supervision, Resources, Writing—Review and Editing. **Ziya Gokaslan:** Supervision, Resources, Writing—Review and Editing. **Patricia Zadnik Sullivan:** Conceptualization, Supervision, Resources, Writing—Review and Editing.

## Ethical approval

This study was approved by the Rhode Island Hospital institutional review board with a waiver of informed consent (IRB #816619).

## Declarations of competing interests

One or more of the authors declare financial or professional relationships on ICMJE-NASSJ disclosure forms.

## Appendix Supplementary materials

Image, application 1
