# Supplementary material for: Metastatic spinal tumor frailty index and New England spinal metastasis score show the most consistent performance for short-term postoperative outcomes: Single-center validation in 114 patients
Source: N Am Spine Soc J. 2026 Apr 25;27:100894. doi: 10.1016/j.xnsj.2026.100894 (PMC13277493; doi:10.1016/j.xnsj.2026.100894)
Supplement: Supplementary file 1 [file mmc1.docx]

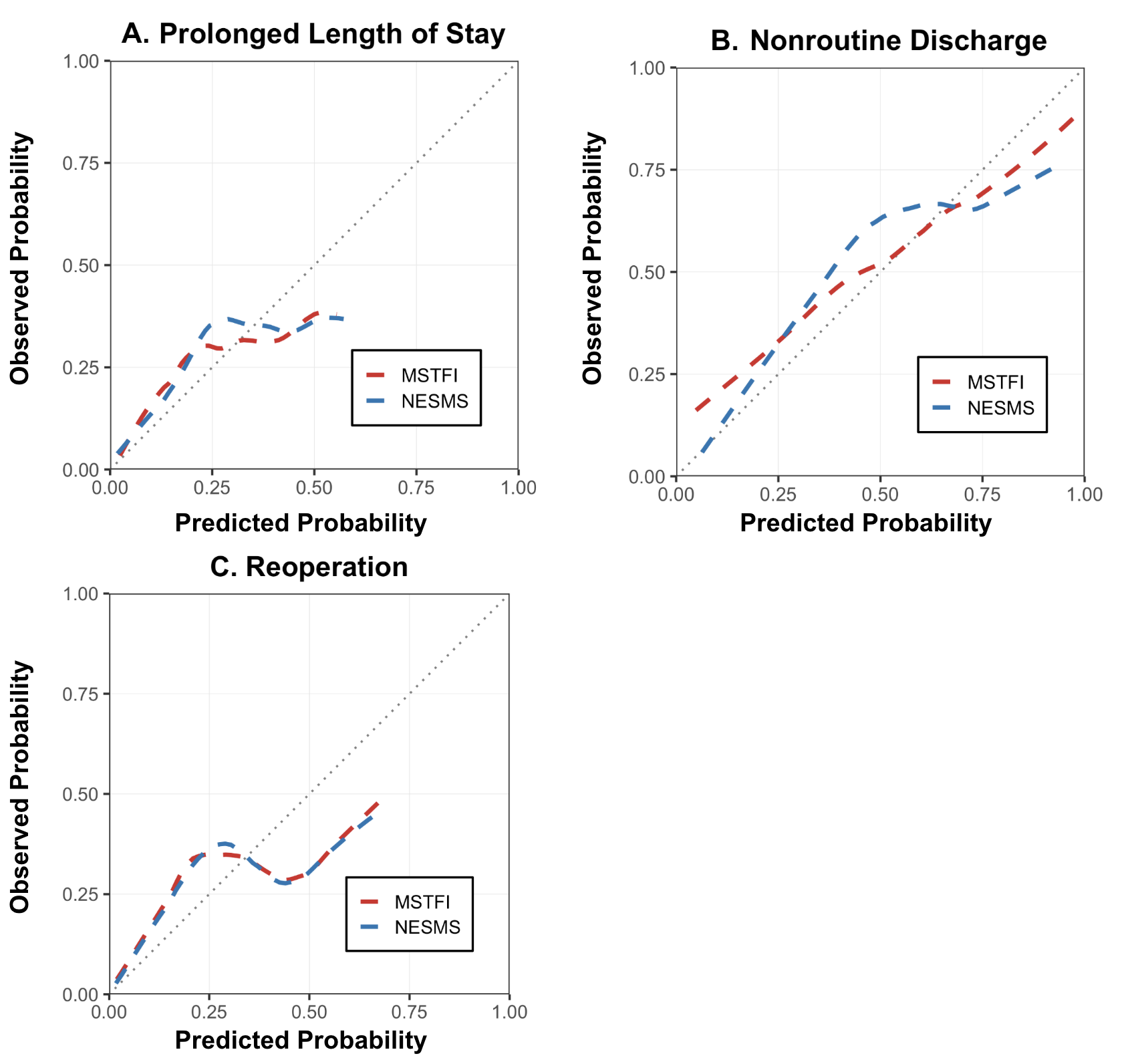


**Supplementary Figure S1.** Calibration plots for (A) prolonged length of stay, (B) nonroutine discharge, and (C) reoperation, comparing the Metastatic Spinal Tumor Frailty Index (MSTFI, red) and New England Spinal Metastasis Score (NESMS, blue). The dashed red and blue lines represent bias-corrected calibration curves from 1,000 bootstrap resamples, and the gray dotted line represents ideal calibration (perfect agreement between predicted and observed probabilities). Calibration was quantified using calibration slope and Brier score.

**
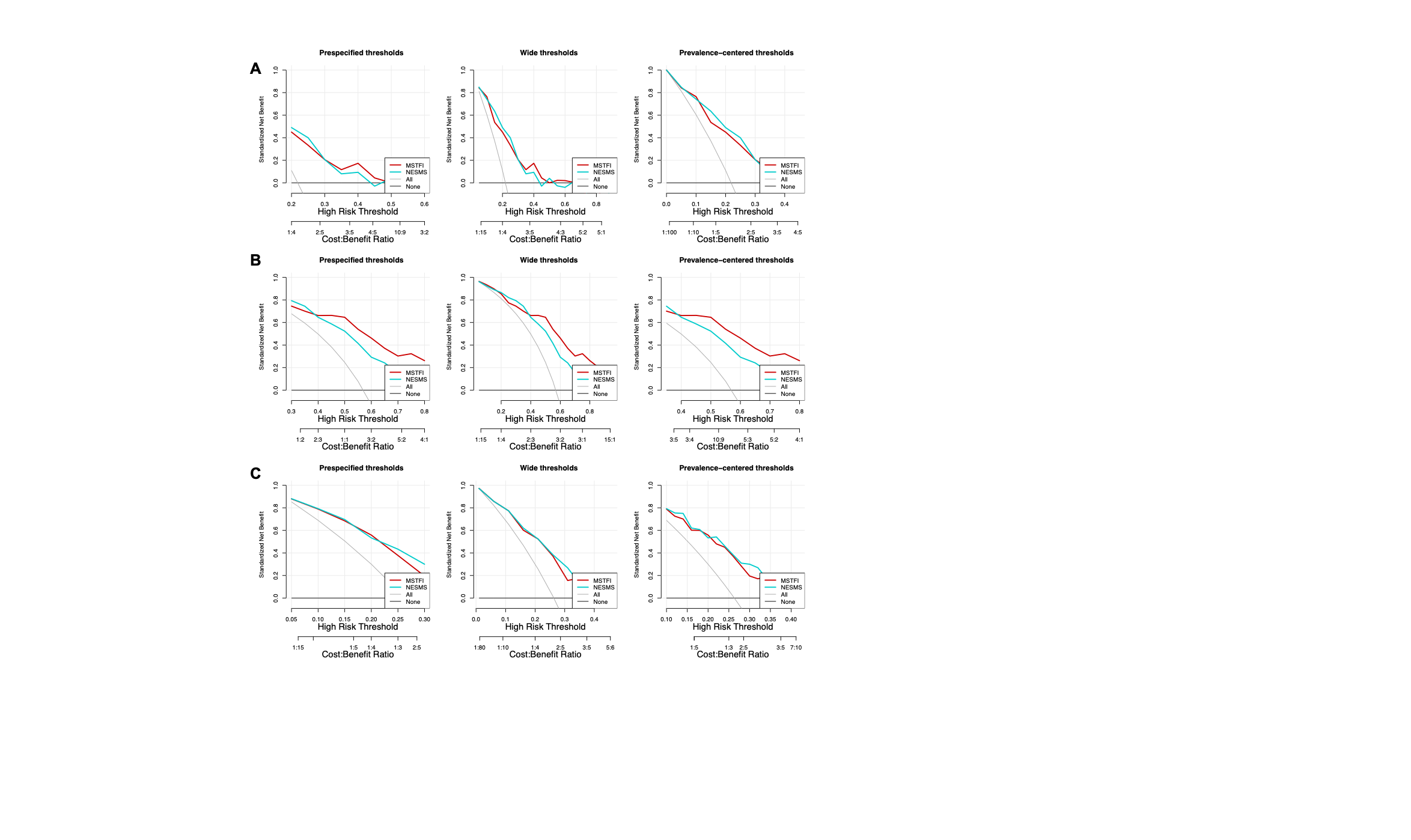
**

**Supplementary Figure S2.** Decision curve analysis comparing the Metastatic Spinal Tumor Frailty Index (MSTFI, red) and New England Spinal Metastasis Score (NESMS, blue) for the primary outcomes (prolonged length of stay, nonroutine discharge, and reoperation) across prespecified and sensitivity threshold probability grids (Supplementary Table S2). The y-axis shows standardized net benefit across high-risk thresholds (with corresponding cost:benefit ratios); gray lines indicate “treat-all” and “treat-none” strategies. Across grids, MSTFI and NESMS generally demonstrated net benefit above reference strategies over plausible threshold ranges.

| **Outcome Variable** | **Events (%)** | **EPV** | **Modeling approach** |
| --- | --- | --- | --- |
| **Primary** |  |  |  |
| Nonroutine discharge | 65 (57.0%) | 6.5 | Standard logistic |
| Prolonged LOS | 25 (21.9%) | 2.5 | Ridge-penalized |
| Reoperation for complication | 19 (16.7%) | 1.9 | Firth-penalized |
| **Secondary** |  |  |  |
| Medical complication | 33 (28.9%) | 3.3 | Standard logistic |
| Spine-related complication | 35 (30.7%) | 3.5 | Standard logistic |
| 30-day ED visit | 39 (34.2%) | 3.9 | Standard logistic |
| Readmission within follow-up | 27 (23.7%) | 2.7 | Ridge-penalized |
| 90-day mortality | 25 (21.9%) | 2.5 | Ridge-penalized |

**Supplementary Table S1.** Events-per-variable (EPV) and modeling approach by outcome.

Events-per-variable (EPV) were calculated as the number of outcome events divided by the number of covariates included in multivariable models. All models included 10 covariates (age, sex, body mass index, hypertension, diabetes, tobacco use, prior radiation, procedure duration, and junctional spinal region, plus index of interest). Standard logistic regression was used for outcomes with higher EPV, ridge-penalized logistic regression for outcomes with limited events, and Firth-penalized logistic regression for rare-event outcomes.

| **Outcome** | **Grid** | **Minimum** | **Maximum** | **Step** | **N** |
| --- | --- | --- | --- | --- | --- |
| Nonroutine discharge | Prespecified | 0.3 | 0.8 | 0.05 | 11 |
| Nonroutine discharge | Wide | 0.05 | 0.95 | 0.05 | 19 |
| Nonroutine discharge | Prevalence-centered | 0.35 | 0.8 | 0.05 | 10 |
| Prolonged LOS | Prespecified | 0.2 | 0.6 | 0.05 | 9 |
| Prolonged LOS | Wide | 0.05 | 0.9 | 0.05 | 18 |
| Prolonged LOS | Prevalence-centered | 0 | 0.45 | 0.05 | 10 |
| Reoperation | Prespecified | 0.05 | 0.3 | 0.05 | 6 |
| Reoperation | Wide | 0.01 | 0.46 | 0.05 | 10 |
| Reoperation | Prevalence-centered | 0.1 | 0.42 | 0.02 | 17 |

**Supplementary Table S2.** Decision curve analysis (DCA) threshold grids used for prespecified and sensitivity analyses. Threshold probability ranges and step sizes applied for each primary outcome (prolonged length of stay, nonroutine discharge, and reoperation), including the prespecified threshold band and the alternative bands (wide, prevalence-centered) used for sensitivity testing. These grids were used to generate the corresponding DCA sensitivity figure (Supplementary Figure S2).
